# Alzheimer's disease: synapses gone cold

**DOI:** 10.1186/1750-1326-6-63

**Published:** 2011-08-26

**Authors:** Robert M Koffie, Bradley T Hyman, Tara L Spires-Jones

**Affiliations:** 1 Massachusetts General Hospital, Harvard Medical School, 114 16th Street, Charlestown, MA 02129, USA; 2 Harvard Biophysics Program, Building C-2, room 122, 240 Longwood Ave, Boston, MA 02115, USA

**Keywords:** Alzheimer's disease, amyloid-beta, synapse loss, long-term depression, long-term potentiation, cognitive decline

## Abstract

Alzheimer's disease (AD) is a progressive neurodegenerative disease characterized by insidious cognitive decline and memory dysfunction. Synapse loss is the best pathological correlate of cognitive decline in AD and mounting evidence suggests that AD is primarily a disease of synaptic dysfunction. Soluble oligomeric forms of amyloid beta (Aβ), the peptide that aggregates to form senile plaques in the brain of AD patients, have been shown to be toxic to neuronal synapses both *in vitro *and *in vivo*. Aβ oligomers inhibit long-term potentiation (LTP) and facilitate long-term depression (LTD), electrophysiological correlates of memory formation. Furthermore, oligomeric Aβ has also been shown to induce synapse loss and cognitive impairment in animals. The molecular underpinnings of these observations are now being elucidated, and may provide clear therapeutic targets for effectively treating the disease. Here, we review recent findings concerning AD pathogenesis with a particular focus on how Aβ impacts synapses.

## Background

First described by the German neuropathologist Alois Alzheimer in 1906, Alzheimer's disease (AD) is a progressive neurodegenerative disease characterized by insidious cognitive decline and loss of memory function [1,2]. Over 35 million people are afflicted with AD worldwide, 5.5 million of them in the United States alone, and these numbers are expected to quadruple by 2050 [3]. AD is the sixth leading cause of death in the United States, and remains one of the only causes of death that increased by as much as 66% over the last decade [4]. No disease-modifying drug has been developed for treating AD, making it one of the most pressing public health problems in the world today. Tremendous progress has been made over the last few decades in understanding the underlying biology of the disease. Here we review pertinent research findings concerning AD pathogenesis with a particular focus on how neuronal synapses are impacted in disease progression. Understanding the molecular underpinnings of AD pathogenesis may aid in developing effective therapeutic approaches for combating it.

### Neuropathology and Pathogenesis of Alzheimer's disease

AD is characterized pathologically by cortical atrophy, neuronal cell death, neuroinflammation, synapse loss, and the accumulation of two definitive pathological lesions: neurofibrillary tangles and senile plaques [5]. Neurofibrillary tangles (NFTs) deposit within neurons and are composed of hyperphosphoryated tau protein whereas senile plaques occur in the extracellular space and are made up largely of the 38-43 amino acid peptide amyloid-beta (Aβ) [6]. Aβ is believed to be a key trigger of AD pathogenesis, one that is upstream of NFTs. It is formed by the sequential cleavage of the amyloid precursor protein (APP) by β- and γ-secretase, after which Aβ is released into the extracellular space [6]. There, Aβ can assume a variety of conformational states ranging from monomers to soluble oligomers, protofibrils, and fibrils, which aggregate to form plaques [7-9].

Several lines of evidence support the hypothesis that alterations in amyloid processing can lead to AD. First, APP is located on chromosome 21, and Down syndrome patients who have trisomy of chromosome 21 invariably develop AD [10]. Further, individuals with trisomy 21 with a chromosome 21q break such that APP diploidy occurs in the setting of trisomy 21 do not develop clinical or neuropathological AD [11]. Conversely, a small cohort of patients who inherited an extra copy of APP due to microduplication of small portions of chromosome 21q containing the APP locus developed AD-like dementia with plaque deposition [12].

Second, most genetic mutations associated with rare familial early onset AD lead to increased production of Aβ or an increase in Aβ42-to-Aβ40 ratio, which increases the propensity for Aβ aggregation [13]. Mutations leading to early onset familial AD have been found in the APP gene on chromosome 21q [14], in the presenilin 1 gene (PSEN 1) on chromosome 14q, and the presenilin 2 gene (PSEN 2, a homolog of PSEN 1) located on chromosome 1q [13]. Presinilin forms the catalytic site of γ-secretase, which is one of the enzymes involved in the cleavage of APP to form Aβ [15-17] All of these mutations influence Aβ metabolism and production [18,19].

Third, Aβ has been shown to be toxic to neurons *in vitro and in vivo *[6]. Injecting synthetic or naturally secreted Aβ, at concentrations akin to those seen in the brains of AD patients, into the brains of rodents induces behavioral deficits and tau hyperphosphorylation [5].

Fourth, transgenic mouse models overexpressing human APP and/or PSEN genes with known familial early onset AD mutations develop amyloid plaque deposition and some of the morphological changes of AD (e.g. synapses loss) [20-22]. While most of these transgenic mice do not develop the typical neuronal cell loss observed in AD, they manifest age-dependent memory impairments and cognitive deficits [20-22].

Finally, immunization of AD transgenic mice with Aβ or anti-Aβ antibodies reduces amyloid plaque deposition, clears existing plaques, and ameliorates cognitive deficits in transgenic mice [23,24], indicating that removal of Aβ is beneficial to the brain.

Taken together, these findings suggest that Aβ is an essential element in the pathogenesis of AD. The mechanistic link between Aβ and neurodegeneration, however, remains elusive. Mounting evidence suggests that AD is primarily a disease of synaptic dysfunction [25] and it is becoming clear that Aβ, particularly in oligomeric form, is toxic to synapses. There is therefore a growing interest in understanding how oligomeric Aβ induces synaptic dysfunction in AD.

### Aβ-mediated synaptic dysfunction in Alzheimer's disease

AD brains are characterized by dramatic synapses loss in mesiotemporal regions [26-29]. Significant synapse loss also occurs in patients with mild cognitive impairment, a harbinger for future AD [30]. In fact, synapse loss is the best pathological correlate of cognitive dysfunction in AD, suggesting that synaptic changes are crucial for AD pathogenesis [28,31,32]. Synapse loss is most prominent in the immediate vicinity of senile plaques, suggesting that plaques may be a reservoir of synaptotoxic molecules such as Aβ [33-36]. Indeed, recent studies using multiphoton *in vivo *imaging revealed a halo of oligomeric Aβ around plaques in the brain of AD transgenic mice suggesting that oligomeric Aβ may exist in equilibrium with plaques in AD [37].

Aβ oligomerizes via an unknown mechanism, adopting several higher order conformations such as soluble dimers, trimers, dodecamers, higher order oligomers (also named Aβ-derived diffusible ligands (ADDL)), protofibrils, and fibrils [38-42]. Most of these higher order Aβ structures have been found to be toxic to neurons. Synthetic Aβ oligomers or natural soluble oligomeric Aβ purified from the media of cultured cells expressing mutant human APP (hAPP) or extracted directly from the brains of AD patients have potent synaptic effects. Sodium dodecyl sulfate (SDS) stable Aβ oligomers, ADDLs and protofibrils [43-47] have all been shown to induce synaptic dysfunction [43-48]. Specifically, oligomeric Aβ inhibits the induction of long-term potentiation (LTP), an electrophysiological correlate of memory formation [41,44,49-53]. Biophysical methods such as size exclusion chromatography (SEC) and mass spectroscopy have been used to show that Aβ dimers and trimers are most potent at inhibiting LTP [50,51]. Inhibitors of Aβ oligomerization rescue impairment of LTP induced by Aβ containing media, suggesting that monomeric Aβ is not a potent inhibitor of LTP [54]. Complementing its effects on LTP inhibition, oligomeric Aβ has also been shown to facilitate the induction of long-term depression (LTD) in hippocampal synapses [52,55,56]. Impairments in LTP and facilitation of LTD culminate in synaptic depression and impairments in neuronal networks [57].

#### Molecular basis of oligomeric Aβ mediated synaptic depression

**Figure 1:**
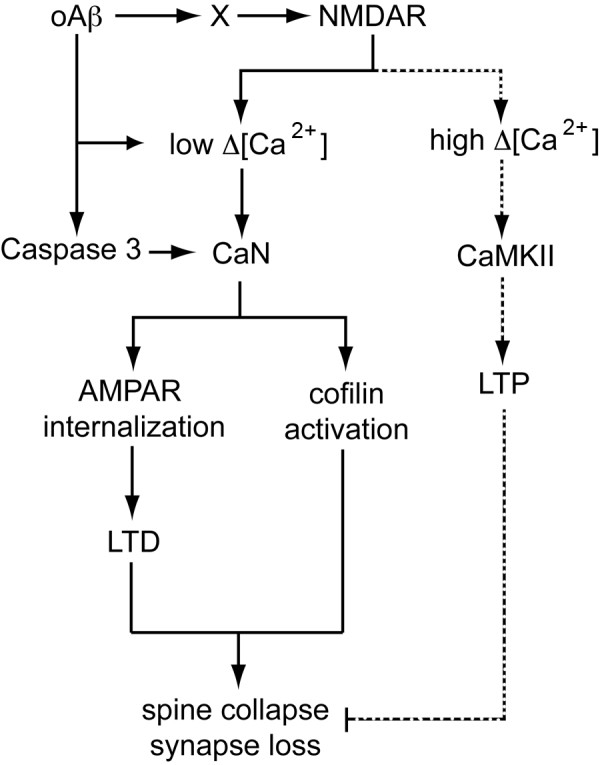
**Molecular pathways of oligomeric Aβ mediated synaptic dysfunction**. Oligomeric Aβ (oAβ) can induce calcium dyshomeostasis, trigger activation of caspase 3, or modulate the activity of NMDARs either directly or through intermediate molecules (shown as X) involved in the trafficking of NMDAR (e.g. EphB2). Activation of different subtypes of NMDA receptors may trigger different intrasynaptic pathways. Activation of NR2A containing NMDARs may lead to high changes in synaptic calcium concentration ([Ca^2+^]), which triggers downstream events involving CaMKII and pCREB (not shown), facilitating the induction of LTP, which promotes dendritic spine enlargement. Alternatively, activation of NR2B containing NMDAR may trigger a low rise in intrasynaptic calcium, which is favored by oAβ interactions with synapses (away from dotted line pathway), leading to calcineurin (CaN) activation; oAβ-dependent active caspase 3 can also activate CaN. Activated CaN dephosphorylates GluR subunits of AMPARs promoting internalization of AMPARs from the surface of synapses leading to LTD, which leads to dendritic spine shrinkage. Furthermore, active CaN dephosphorylates cofilin rendering it active to depolymerize dendritic spine actin, which leads to dendritic spine collapse and synapse loss.

The molecular mechanisms underlying oligomeric Aβ-mediated synapse dysfunction is very complex. Oligomeric Aβ can induce calcium dyshomeostasis, trigger activation of caspases and calcineurin, and modulate the activity of synaptic excitatory receptors and receptor tyrosine kinases, instigating a cascade of molecular events that culminate in the inhibition of LTP, facilitation of LTD, and synapse loss (Figure 1).

Physiologically, LTP and LTD depend on calcium influx through N-methyl-D-aspartate (NMDA) receptors and/or activation of metabotropic glutamate receptors (mGluRs) [58-62]. Synapse potentiation or depression depends on the rate of influx of calcium as well as the level of cytosolic calcium. LTP occurs when rapid and high levels of calcium influx occur through NMDA receptors, whereas LTD is favored when low level calcium influx through NMDA receptors occurs [63]. LTP requires the activation of NR2A containing NMDA receptors, whereas LTD requires activation of NR2B containing NMDA receptors [64]. These different subclasses of NMDA receptors have distinct calcium influx kinetics [65,66] and modulate distinct postsynaptic signaling pathways [67,68]. LTP is associated with dendritic spine enlargement and increase in synapse density, whereas LTD leads to dendritic spine shrinkage and synapse collapse [69-72]. Several protein kinases such as p38 mitogen-activated protein kinase (MAPK), calcium calmodoulin-dependent protein kinase II (CaMKII), glycogen synthase kinase 3-beta (GSK3β), and ephrin receptor B2 (EphB2) have all been shown to modulate LTP induction in the brain [73,74]. Phosphatases and proteases such as calcineurin (protein phosphatase 2B [PP2B]) and caspases play key intracellular roles in the induction of LTD [58,62,75]. Transcription factors such as the cyclic AMP response element binding protein (CREB) are crucial for the induction of continuous LTP, by increasing the expression of several genes including those encoding brain derive neurotrophic factor (BDNF) and nitric oxide synthase [76,77].

Oligomeric Aβ has been shown to inhibit LTP and enhance LTD by modulating the activity of all of the above molecular pathways. Oligomeric Aβ-induced loss of excitatory synapses in the hippocampus requires functional NMDA receptors [51]. Several studies have shown that oligomeric Aβ induces partial blockade of NMDA receptor currents, which leads to reduction of calcium influx into spines promoting LTD over LTP [78,79]. Aβ binds to 7α-nicotinic acetylcholine receptors (nAchR) [80], triggering a series of events that leads to internalization of NMDA receptors via a mechanism requiring calcineurin activation [81]. Reduced calcium influx through NMDA receptors induced by Aβ limits CAMKII function, LTP, and spine enlargement [82]. In fact, oligomeric Aβ-mediated LTP impairment is believed to involve a decrease in the activation of MAPK, CaMKII and Akt/protein kinase B, but not protein kinases A and C [53,83,84]. Aβ has also been shown to induce synaptic depression by activating mGluRs, which triggers a series of downstream molecular events involving MAPK and calcineurin, ultimately promoting internalization of α-amino-3-hydroxy-5-methyl-4-isoxazolepropionic acid (AMPA) receptors and synapse collapse [73,85]. *In vivo *studies suggest that Aβ indirectly modulates calcineurin activation by causing calcium dysregulation [86-88]. Calcineurin activation promotes the induction of LTD by decreasing surface expression of NMDA receptors and increasing internalization of AMPA receptors via dynamin-mediated endocytosis [79,89]. Indeed, Aβ-mediated internalization of AMPA [85] and NMDA receptors [81], loss of dendritic spines [85], and cognitive decline [90] can all be rescued by inhibiting calcineurin activation [91-93], indicating that calcineurin plays a crucial role in Aβ-dependent modulation of synaptic plasticity. Further, oligomeric Aβ activation of calcineurin has been shown to induce dendritic simplification, spine loss, and neuritic dystrophies at least in part by activating NFAT (nuclear factor of activated T-cells) pathways both *in vitro *and *in vivo *[91]. Oligomeric Aβ has also been shown to activate other synaptic phosphatases such as STEP (striatal-enriched tyrosine phosphatase), which function to dephosphorylate NR2B subunits of NMDA receptors and promote their endocytosis, thereby inducing synaptic depression [94-96].

Oligomeric Aβ can also directly interact with synaptic surface receptor tyrosine kinases that play key roles in LTP and LTD modulation. For instance, it has been shown that oligomeric Aβ binds to the fibronectin domain of EphB2, a receptor tyrosine kinase known to modulate NMDA receptor trafficking and downstream transcription factors such as Fos, which plays a critical role in the induction of LTP [97-100]. Oligomeric Aβ binding to EphB2 promotes its degradation in the proteasome, impairing the induction of LTP [101]. Indeed, EphB2 is depleted in the brains of transgenic hAPP mice and AD patients [102], and replacement of EphB2 reverses cognitive impairment in hAPP mice [101].

Other studies have shown that Aβ facilitates hippocampal LTD via a mechanism that depends on both NMDAR and mGluR activity. Exogenous extracellular glutamate scavengers reverse oligomeric Aβ mediated facilitation of LTD, whereas inhibitors of glutamate reuptake mimic oligomeric Aβ-mediated LTD facilitation, suggesting that the effects of oligomeric Aβ-mediated LTD facilitation may occur as a result of impaired glutamate reuptake at the synapse, leading to post-synaptic NMDA receptor desensitization [55]. Metabotropic glutamate receptor activity, GSK-3β signaling, and protein phosphatase 2B activity are all necessary for oligomeric-Aβ mediated LTD enhancement [55,73].

Caspase-3 activity has also been found to be crucial for oligomeric Aβ-mediated facilitation of LTD. Soluble Aβ induces caspase-3 activation at a low level that is not sufficient to induce apoptosis [84]. Mitochondria-dependent caspase-3 activation is necessary for physiologic LTD via a mechanism involving Akt proteolysis [75]. Soluble Aβ activates caspase-3, which leads to LTD via a mechanism involving activation of different protein phosphatases that dephosphorylate AMPA receptors and promote their endocytosis from synaptic surfaces, suggesting that prevention of caspase-3 activation may be a viable therapeutic approach for treating AD [84]. Acute inhibition of caspase-3 activity is beneficial, but unfortunately, chronic inhibition of caspase-3 activation beyond the baseline did not reverse cognitive decline in hAPP mice, but instead exacerbated cognitive impairment, possibly due to a requirement for caspase-3 activity in normal synaptic function [84]. Aβ also influences CREB activation, which is crucial for the maintenance of LTP, insofar as CREB regulates the expression of genes necessary for LTP. One study showed that Aβ decreases the activity of CREB and thus reduces expression of genes encoding proteins that are essential for LTP [103]. Another study found that excessive activation of extrasynaptic NR2B-containing NMDA receptors, which leads to downregulation of CREB underlies oligomeric Aβ-mediated LTP inhibition [104].

#### Oligomeric Aβ causes synapse shrinkage in Alzheimer's disease

The acute effects of Aβ on synaptic physiology appear to translate into structural changes in synaptic morphology because enhanced LTD leads to dendritic spine shrinkage whereas inhibition of LTP limits spine enlargement [69-72]. Exposure of cultured neurons or rat hippocampal slices to oligomeric Aβ induces dendritic spine shrinkage and collapse, a phenomenon that can be reversed by treatment with Aβ antibodies [51,105]. APP transgenic mice have significant synapse loss and neutralization of oligomeric Aβ with anti-Aβ antibodies leads to reversal of synapse collapse [106-108]. Furthermore, increased concentration of Aβ may reduce glutamatergic transmission and leads to synapse loss in hAPP transgenic mice even before plaque formation [21,109,110]. Oligomeric Aβ-mediated inhibition of LTP and enhancement of LTD lead to dendritic spine loss as a result of F-actin remodeling [105]. LTD accompanied by shrinkage of dendritic spines occurs via a mechanism involving cofilin-mediated depolymerization of actin [71]. Specifically, Aβ indirectly stimulates cofilin binding to actin and induction of actin depolymerization in neuronal cytoskeleton. Binding of cofilin to actin is promoted by dephosphorylation at Ser3 by phosphatase Slingshot, and inhibited by phosphorylation by LIM kinase 1, a process that is modulated by oligomeric Aβ [105]. Indeed, in addition to dendritic spine protein loss, increased amounts of dephosphorylated cofilin have been found in the brain of AD patients [111,112].

#### Oligomeric Aβ induces cognitive impairments

The electrochemical and structural effects of oligomeric Aβ on synapses described above may lead to potent behavioral and cognitive deficits in animals. Intra-cerebral injection of synthetic or naturally secreted oligomeric Aβ impairs complex behavior including memory and cognitive function in animals [113-115]. APP transgenic mice with increased soluble Aβ in the brain display dramatic cognitive impairments even before the onset of plaque deposition [21]. Neutralization of soluble oligomeric Aβ with anti-Aβ antibodies reverses behavioral deficits seen in different AD transgenic mice [116-118], suggesting that behavioral deficits in AD transgenic mice are caused by soluble Aβ. Inhibition of oligomeric Aβ formation decreases both histopathological and behavioral AD phenotypes in APP transgenic mice [119], implicating higher order Aβ structures such as soluble oligomeric Aβ, but not Aβ monomers, in AD pathogenesis. Levels of soluble oligomeric Aβ, but not senile plaques, in the brain correlates with severity of memory loss in human AD patients, however, the precise contribution of different Aβ species to cognitive decline is not clear [120].

While it is now well-established that increased oligomeric Aβ levels in the brain leads to synaptic dysfunction, it should be noted that at physiologic levels, Aβ might play a normal role in modulating synaptic activity, which likely becomes deranged in the setting of excess Aβ production or accumulation, leading ultimately to the clinical manifestation of cognitive impairment. Indeed, there is a small but growing body of evidence suggesting that Aβ at low concentrations actually promotes LTP and normal synaptic function [121-124]. Thus, therapeutic approaches aimed at improving cognition by counteracting the toxic effects of Aβ will have to be tailored to target only the toxic function of oligomeric Aβ. Nonspecific total inhibition of Aβ may lead to negative effects on synaptic function and cognition.

### Seeing Aβ in action at synapses

**Figure 2:**
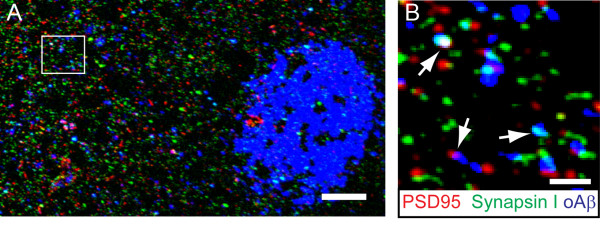
**Oligomeric Aβ associates with a subset of synapses in the brain of Alzheimer's disease transgenic mice**. **A**) Array tomograms showing oligomeric Aβ (oAβ) localized to synaptic sites near senile plaques in APP/PS1 mice. **B**) A higher magnification view of the outlined square in panel **A **showing multiple pre- and post-synaptic elements colocalized with oAβ (arrows) stained with an oAβ specific antibody (NAB61). Scale bar is 10 μm in **A **and 2 μm in **B**.

Collectively, all of the above evidence suggests that soluble oligomeric Aβ is a potent mediator of cognitive impairment in AD. Oligomeric Aβ inhibits the induction of LTP, lowers the threshold for inducing LTD, and causes synapse collapse, which may ultimately lead to cognitive decline resulting from disrupted neuronal network connectivity [57]. For several years, limitations in the resolution of conventional microscopy techniques made it difficult to ascertain whether oligomeric Aβ directly associates with neuronal synapses and plays a role in their shrinkage and collapse *in vivo*. Recent advances in high-resolution microscopy techniques have made it possible to address these questions. For example development of array tomography [125,126], an ultra-high resolution fluorescence imaging technique that allow direct simultaneous visualization of several thousand small structures such as synapses and peptides in tissue has allowed determination of whether oligomeric Aβ plays a direct role in synapse loss in AD. Using array tomography and a conformation specific antibody (NAB61) [127], we demonstrated that oligomeric Aβ in the brain of APP/PS1 transgenic mice directly colocalizes with a subset of synapses and is associated with their shrinkage and collapse [37] (Figure 2), suggesting that the *in vitro *effects of Aβ oligomers observed using cell based assays likely also occur *in vivo*, supporting the notion that oligomeric Aβ adversely impacts synapses. High-resolution techniques such as array tomography could be extended to study the effects of oligomeric Aβ on synapses in the brain of AD patients. Furthermore, it will be important to determine whether Aβ oligomers are targeted to synapses by specific carrier proteins or whether they are produced locally at synapses. A number of studies have suggested that production of Aβ (at least in monomeric form) is regulated by activity [110,128-130] and Aβ appears to play a negative feedback function on synaptic activity [110,131,132]. Mechanistically, synaptic activity-dependent production of Aβ requires clathrin-mediated endocytosis of APP, which is then cleaved by β- and γ-secretase in late endosomes at synapses to form Aβ [129]. Nonetheless, it is also possible that Aβ binding proteins like apolipoprotein E, which also play a role at the synapse, may stabilize Aβ oligomers [133] in the extracellular space and deliver them to synaptic sites.

It is presently unclear whether Aβ oligomers interact directly with specific high affinity receptors at the synapse to induce synapse dysfunction. A number of recent studies have reported high affinity binding of oligomeric Aβ to cellular prion protein (PrP^C^), which was necessary for Aβ to mediate acute synaptic depression, synapse loss, and cognitive impairment *in vivo *[134,135]. Subsequent studies, however, could not reproduce these findings [136-138]. This is likely because of differences in experimental paradigms used in the subsequent studies. Single particle tracking of Aβ oligomers labeled with quantum dots exposed to hippocampal neurons in culture have nonetheless demonstrated that the diffusion of Aβ oligomers is dramatically limited upon binding to synaptic sites, suggesting that high affinity oligomeric Aβ receptors may be present at synapses [139]. Identifying these high affinity receptors could aid in designing drugs capable of blocking the deleterious effects of oligomeric Aβ on neuronal synapses.

### Concluding remarks

Based on the evidence discussed here, we postulate that AD begins as a disease of synaptic dysfunction and synapse loss then progresses to include widespread neuronal loss and neuronal network failure. Findings from recent experiments continue to provide insight into the complicated molecular underpinnings of synapse dysfunction in AD with mounting evidence pointing to soluble oligomeric Aβ as a key player in the induction of synaptic failure. Oligomeric Aβ activates a variety of molecular cascades that culminate in synapse dysfunction, shrinkage, collapse and loss (Figure 1). These pathological Aβ-triggered molecular events, however, may become independent of Aβ as the disease progresses, with downstream tau effects causing overt neuronal loss, exacerbating the loss of connectivity between neurons [140]. If this is correct, at least two main therapeutic approaches could be taken to combat the disease effectively: 1) early interventions that prevent the initiation of Aβ-triggered pathological events; or 2) inhibition of specific downstream pathways activated by Aβ. The failure of previous therapeutic approaches aimed at removing toxic Aβ species from the brain (e.g. active immunization with Aβ peptide) in clinical trials may be because they were given to the wrong cohort of patients (i.e. patients with advanced AD, whose Aβ-triggered neuronal events may have become independent of Aβ) [140]. Perhaps, a more effective approach will be to initiate such anti-Aβ therapeutic regimens at very early stages of the disease. For this approach to be successful, highly sensitive and specific biomarkers for diagnosing AD need to be developed to identify AD patients at the very early stages of the disease. For patients who have progressed into symptomatic AD, it will likely be necessary to target pathways downstream of Aβ, including tau hyperphosphorylation and accumulation in the soma, which are linked to neuronal death [141,142]. In conclusion, Aβ-mediated synaptic dysfunction appears to be an important driving factor in AD pathogenesis and understanding the molecular underpinnings may provide effective therapeutic targets for combating the disease.

## Competing interests

The authors declare that they have no competing interests.

## Authors' contributions

RMK designed the layout and content of the review; RMK collected illustrative data, RMK, BTH, and TLS-J wrote the text; all authors read and approved the final manuscript.
